# Prevalence of fatty pancreas and its relation with anthropometric values on the Growth and Obesity Cohort Study

**DOI:** 10.1016/j.jped.2024.09.007

**Published:** 2024-12-07

**Authors:** Gigliola Alberti, Thelma Cantillo, Ana Pereira, Florencia De Barbieri, Cristian García, Luis Villarroel, Juan Cristóbal Gana

**Affiliations:** a Department of Pediatric Gastroenterology and Nutrition, Division of Pediatrics, Escuela de Medicina, Pontificia Universidad Católica de Chile, Santiago, Chile; b Pontificia Universidad Católica de Chile, Santiago, Chile; c Instituto de Nutrición y Tecnología de los Alimentos (INTA), Universidad de Chile, Santiago, Chile; d Radiology Department, Escuela de Medicina, Pontificia Universidad Católica de Chile, Santiago, Chile; e Department of Public Health, Escuela de Medicina, Pontificia Universidad Católica de Chile, Santiago, Chile

**Keywords:** Nonalcoholic fatty pancreas disease, Pancreatic steatosis, Adolescence

## Abstract

**Objective:**

Nonalcoholic Fatty Pancreas Disease (NAFPD) is characterized by excessive lipid accumulation within the pancreas in the absence of alcohol intake, potentially leading to pancreatic dysfunction and metabolic complications, including type 2 diabetes mellitus, acute and chronic pancreatitis, and pancreatic carcinoma. The authors aim to estimate the prevalence of NAFPD and its association with anthropometric parameters in a cohort of Chilean adolescents.

**Method:**

The authors conducted a cross-sectional analysis of the "Growth and Obesity Chilean Cohort Study" (GOCS), a longitudinal study involving nearly 1000 children, followed yearly since 2006. All participants underwent anthropometric measurements and abdominal ultrasonography.

**Results:**

A total of 741 adolescents were included; 30 exhibited ultrasonography findings compatible with fatty pancreas (4 %). Adolescents with NAFPD had higher BMI z-score (2.33 (1.52–2.69) vs 0.67 (-0.2–1.4), *p* < 0.001), waist circumference (WC) (90.9 (81.53–98.58) vs 72.2 (67.55–79.83), *p* < 0.001), waist-to-height ratio (0.55 (0.48–0.6) vs 0.44 (0.41–0.49), *p* < 0.001), triponderal index (17.35 (15.14–19.25) vs 13.62 (12.07–15.54), *p* < 0.001), subcutaneous fat (32.4 (21.77–44.95) vs 16.2 (9.3 - 25.3), *p* < 0.001), visceral fat (45.15 (36.92–62.08) vs 35.5 (28.55–44.25), *p* < 0.001), systolic blood pressure (*p* = 0.009), and diastolic blood pressure but only in boys (*p* = 0.004) compared with controls. The prevalence of liver steatosis was significantly higher in the NAFPD group (63.3% vs 5.2 %, *p* < 0.001). After adjusting for sex and BMI, only the association with waist circumference and liver steatosis remains statistically significant.

**Conclusion:**

In adolescents, NAFPD has a prevalence of 4 % and is associated with a higher BMI z-score, WC, superficial fat, and blood pressure levels. Liver steatosis exhibited a strong association with NAFPD.

## Introduction

Obesity has become a significant global health challenge, playing a key role in the escalating prevalence of chronic non-communicable diseases. According to the World Health Organization (WHO), the worldwide prevalence of obesity has nearly tripled over the last 40 years. Specifically, the prevalence of overweight and obesity among children and adolescents aged 5–19 has surged from 4 % in 1975 to over 18 % in 2016.^1^ In Chile, adolescent overweight and obesity prevalence saw a 50.3 % increase in 2022.^2^

Obesity, especially central obesity, induces ectopic fat accumulation in various organs such as the liver, heart, and pancreas, leading to a pro-inflammatory state. Fatty pancreas (FP) or non-alcoholic fatty pancreas disease (NAFPD) involves excessive lipid accumulation in the pancreas without alcohol intake, viral infections, toxins, or congenital metabolic syndromes.^3^^,^^4^ NAFPD was initially described by Ogilvie in 1933 in individuals with obesity.^5^ In 2010, van Geenen et al.^6^ suggested that obesity, particularly its association with insulin resistance, plays a crucial role in adipocyte infiltration into the pancreas. Analogous to liver steatosis (LS), NAFPD clinically ranges from simple fat deposition to pancreatic inflammation and fibrosis.^7^ The main pathogenic mechanism of NAFPD involves fat accumulation within the pancreas, either intralobular or interlobular, leading to dysfunction. Excessive weight gain causes fat to accumulate in both acinar and islet cells, resulting in cell death and replacement by adipocytes. Additionally, fat deposits around large vessels and ducts activate pancreatic stellate cells, contributing to fibrosis. These changes impair insulin secretion and β-cell function, potentially leading to conditions like diabetes.^8^

Despite the global prevalence of NAFPD and its association with obesity, its occurrence in adolescence remains unknown, and its true clinical impact is unclear. Human studies have linked FP with type 2 diabetes mellitus, acute and chronic pancreatitis, pancreatic carcinoma (PC), LS, and atherosclerotic markers. A recently published systematic review found that 32 % of patients with FP had PC (OR 1.32, 95 % CI 0.42–4.16), and the likelihood of having FP among patients with PC was over six times higher (OR 6.13, 95 % CI 2.61–14.42) than in those without PC, suggesting that FP could be a significant risk factor for PC.^9^ Additionally, pancreatic fatty infiltration correlates with metabolic risk factors, potentially serving as a significant manifestation of metabolic syndrome.

It is imperative to determine the authentic prevalence of NAFPD in the adolescent population and proactively identify the disease in its early stages to prevent its progression into metabolic or tumoral pathologies. The primary objective of this research is to examine the frequency of NAFPD occurrence in a well-characterized cohort of Chilean adolescents and its correlation with anthropometric parameters and adiposity markers.

## Methods

### Participants

Cross-sectional study within the Chilean Growth and Obesity Cohort Study (GOCS), an ongoing longitudinal study initiated in 2006. Children born between 2002 and 2003, attending public schools in Santiago, were invited to participate if they met specific criteria: single birth, birth weight between 2500 and 4500 g, and no physical or psychological conditions that could impact their growth. A total of 1190 children were recruited and assessed annually since 2006. The GOCS participants were representative of the general population regarding gender, socioeconomic status, and anthropometric measurements at birth.^10^ For this study, 784 adolescents underwent evaluation between 2016 and 2019 to determine the presence of NAFPD.

Participants with any of the following conditions were excluded: a previous history of acute or chronic pancreas disease or chronic liver disease, significant alcohol consumption (over 20 g/day), and the presence of malignant disease or severe health conditions that could interfere with the study's results.

### Anthropometric assessment

Weight and height were obtained using a digital weight scale (TANITA 418 BCE, 0.1 Kg precision) and a portable stadiometer (SECA 222, 0.1 cm precision), respectively. Body mass index (BMI) was calculated as the ratio of weight (in kg) to the square of height (in meters). BMI-for-age (BMI z-score) was determined using the WHO 2007 growth reference.^11^ Classification included normal weight for BMI-z scores between −1 and 1 SD, overweight for BMI-z scores greater than 1 SD up to 2 SD, and obesity for BMI-z scores exceeding 2 SD.

Waist circumference (WC) was measured using an inextensible metal tape measure (W606PM model; Lufkin, 0.1 cm precision), taken just above the iliac crest at the end of a normal expiration. The waist-height ratio (WHtR) was calculated by dividing the waist by height, in centimeters. The triponderal mass index (TPI) was calculated as weight (kg) divided by height (m) cubed. Blood pressure (BP) was assessed utilizing the OMRON 705‐IT digital sphygmomanometer, model LUFKIN W606PM. Participants were seated with their arms resting on a table after a minimum of 10 min. Four BP readings were taken, with the initial reading excluded, and the average of the subsequent three readings was used to determine systolic and diastolic BP.

### Diagnosis of NAFPD and LS

Transabdominal ultrasound (US) was performed using an Acuson S-2000 unit with 6–2 MHz convex and 9–4 MHz linear transducers by two pediatric radiologists. The diagnosis of NAFPD was established when the echogenicity of the pancreatic parenchyma exceeded that of the adjacent liver (in the absence of fatty liver) or the renal cortex (in the presence of fatty liver).^12^ Liver steatosis was diagnosed based on the echogenicity of the liver in comparison with the renal cortex.^13^ Additionally, the thickness of subcutaneous and visceral abdominal fat was measured with the US at the supraumbilical region using a previously established method.^14^ One radiologist performed and reported findings for half of the cohort, while the other radiologist conducted and reported findings for the remaining half of the cohort.

### Statistical analysis

The participants in the study were categorized into two groups: the cases group, consisting of individuals with NAFPD, and the control group, comprising those individuals without NAFPD. Anthropometric characteristics were summarized using mean, standard deviation, median, and interquartile range for continuous variables. To compare continuous variables, the authors employed Wilcoxon's rank test and reported corresponding p-values.

Crude and adjusted logistic models were performed to estimate the odds ratio (OR) and its 95 % confidence intervals (95 % CI) for each studied anthropometric measure and NAFPD. These models were adjusted for potential confounders, such as age (years) and sex. Additionally, to assess the association of fat distribution measures (WC, WHtR, TPI) with NAFPD independent of BMI, the logistic regression models were further adjusted for BMI. This approach ensured a comprehensive examination of the association between anthropometric measures and NAFPD, considering potential confounders and the impact of BMI on fat distribution measures.

### Ethics

The Ethics committee of the School of Medicine of the Pontificia Universidad Católica de Chile (ID: 200312012) and of the Institute of Nutrition and Food Technology (INTA) of the Universidad de Chile approved the protocol and the informed consent used in the study. Signed informed consent and assent were obtained prior to the enrollment from the parents and children, respectively.

## Results

### Characteristics of the participants

**Figure 1:**
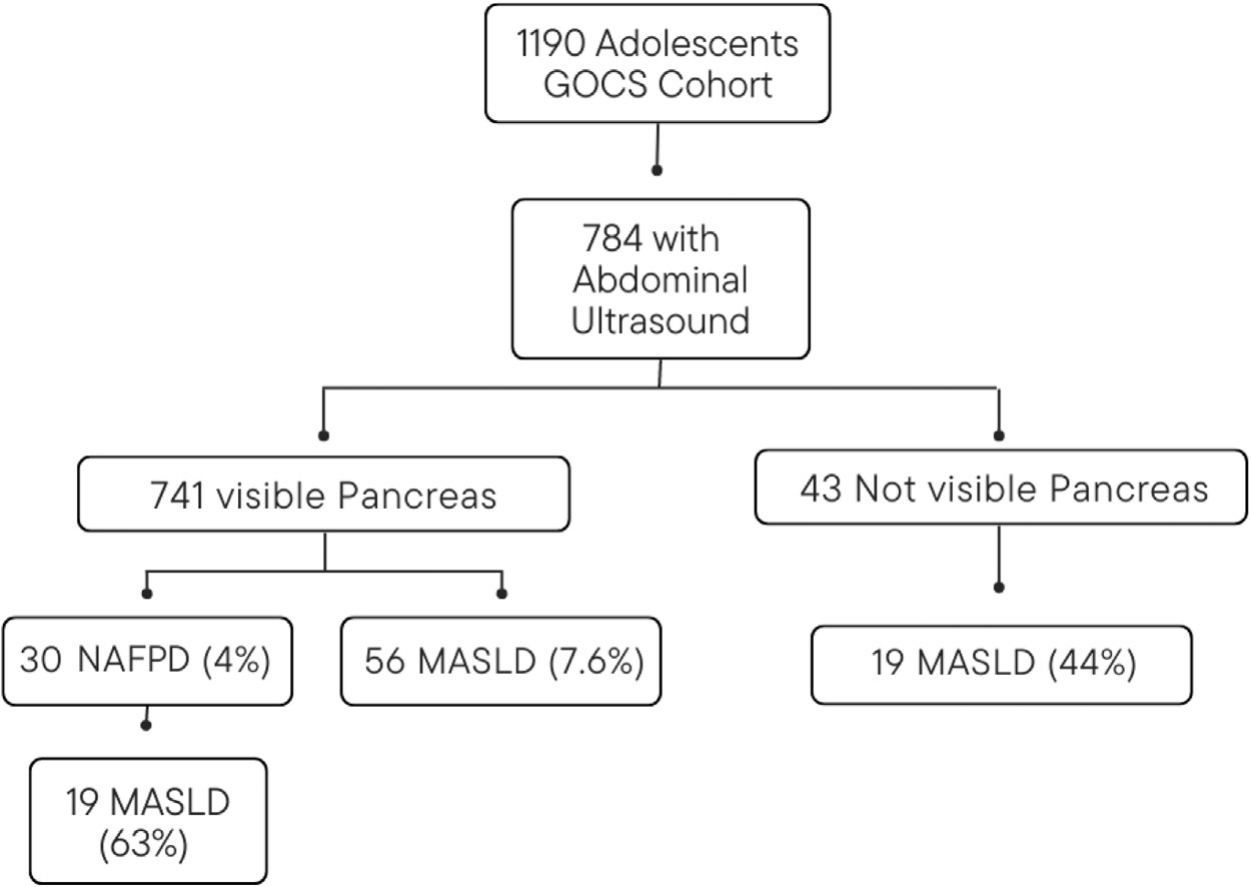
Flowchart of participants.

A total of 784 adolescents were assessed, and successful pancreas visualization was achieved in 741 participants (Figure 1). The mean age of the participants was 15.43 years (SD ± 0.97, range 13.2 to 17.9), with 49.1 % males. Table 1 shows the general characteristics of the participants. In the sample analyzed, the percentage distribution of each nutritional status was as follows: obesity 12.4 % (severe obesity 1.5 %), overweight 27.2 %, underweight 6 %, and normal nutritional status 54.4 %. Out of the total participants, 30 (4 %) exhibited NAFPD, and 56 (7.6 %) had LS. Among the participants with NAFPD, 19 (63.3 %) also presented LS.

**Table 1:** General characteristics of the participants.

Variables (*n* = 741)	Mean ± SD
Age, years	15,43 ± 0,97
Sex, N (%)	
Male	364 (%)
Female	377 (%)
Weight, kg	61,67 ± 13,56
Height, cm	163,6 ± 7,8
BMI, kg/m2	23,03 ± 4,64
z - BMI	0,79 ± 1,94
WC, cm	74,89 ± 10,8
WHtR	0,46 ± 0,065
TPI	14,12 ± 2,97
Subcutaneous fat, mm	19,04 ± 13,16
Visceral fat, mm	37,42 ± 12,41
Fatty pancreas, n (%)	30 (4 %)
Liver Steatosis, n (%)	56 (7.6 %)

BMI, body mass index; z-BMI, body mass index z score; WC, waist circumference; WHtR, weight to height ratio; TPI, Triponderal mass index; SBP, systolic blood pressure; DBP, diastolic blood pressure.

### Characteristics of the groups with and without fatty pancreas

Table 2 compares the cohort characteristics between the cases and controls. Regarding age, participants in the FP group had a median age of 14.77 years (IQR 14.36–15.74), slightly lower than the mean age of 15.38 years (IQR 14.66–16.27) in the control group. This age difference between the two groups was statistically significant (*p* = 0.021). In terms of sex distribution, the NAFPD group comprised 18 males and 12 females, while the control group had 346 males and 365 females. The difference in sex distribution between the two groups was not statistically significant (*p* = 0.2649).

**Table 2:** Characteristics of the groups with and without fatty pancreas.

Variable	FP (30)	Controls (711)	*p-* value	Male FP (18)	Male controls (346)	*p*-value	Female FP (12)	Female controls (365)	*p-*value
Age (years)	14.77 (14.36–15.74)	15.38 (14.66–16.27)	**0.021**	14.49 (14.22–15.12)	14.82 (14.37–15.61)	0.154	15.57 (15.03–16.14)	15.87 (15.2–16.45)	0.166
Sex (Male/Female)	18/12	346/365	0.2649						
Weight, Kg	78.83 (66.85–90.34)	58.7 (52.83–67.45)	**<0.001**	78.5 (65.58–91.65)	59.15 (52.9–68.45)	**<0.001**	78.97 (70.12–85.09)	58 (52.8–65.6)	**<0.001**
Height, cm	166.82 (160.95–171.35)	163.15 (158.18–168.82)	0.061	170.2 (167.72–173.4)	168.4 (163.85–172.39)	0.159	161.05 (155.39–164.14)	158.65 (154.50- 162.45)	0.401
BMI, kg/m2	28.47 (25.21–31.43)	22.15 (19.86–25.08)	**<0.001**	27.6 (24.36–30.4)	20.88 (18.99–23.97)	**<0.001**	30.4 (27.02–35.68)	23.14 (20.94–25.77)	**<0.001**
z- BMI	2.33 (1.52–2.69)	0.67 (−0.12–1.4)	**<0.001**	2.34 (1.52–2.59)	0.44 (−0.35–1.31)	**<0.001**	2.25 (1.84–2.95)	0.83 (0.14–1.48)	**<0.001**
WC, cm	90.9 (81.53–98.58)	72.2 (67.55–79.83)	**<0.001**	89.38 (82.09–99.66)	72.03 (68.1–79.1)	**<0.001**	90.9 (78.06–96.26)	72.55 (67.25–80.2)	**<0.001**
WHtR	0.55 (0.48–0.6)	0.44 (0.41–0.49)	**<0.001**	0.54 (0.49–0.57)	0.43 (0.41–0.47)	**<0.001**	0.56 (0.48–0.61)	0.46 (0.42–0.5)	**<0.001**
TPI	17.35 (15.14–19.25)	13.62 (12.07–15.54)	**<0.001**	16.38 (14.44–17.88)	12.42 (11.3–14.12)	**<0.001**	18.67 (16.97–22.87)	14.61 (13.17–16.33)	**0.001**
SBP, mmHg	114.33 (105.67–122)	109 (102–115.67)	**0.009**	117.67 (110.67–123)	110.67 (104.33–117)	**0.002**	104.83 (103.33–114.5)	107.33 (100.67–114.17)	0.773
DBP, mmHg	64.33 (59.67–70.67)	62.67 (57–67.67)	0.059	64.33 (61.33–71)	61 (55.67–65.42)	**0.004**	63.5 (58.67–69.67)	64.33 (59.33–69.33)	0.999
Subcutaneous fat, mm	32.4 (21.77–44.95)	16.2 (9.3–25.3)	**<0.001**	29.75 (21.25–36.15)	10.45 (6–18.48)	**<0.001**	38.6 (24.25–49.88)	21.5 (14.6–29.6)	**0.002**
Visceral fat, mm	45.15 (36.92–62.08)	35.5 (28.55–44.25)	**<0.001**	44.6 (35.62–53.55)	36.9 (30.45–44.95)	**0.038**	51.7 (37.3–67.12)	34.85 (26.88–43.6)	**0.001**
Liver Steatosis	19 (63.3 %)	37 (5.2 %)	**<0.001**	11 (61.1 %)	13 (3.7 %)	**<0.001**	8 (66.6 %)	24 (6.6 %)	**<0.001**

The data is presented in the median and interquartile range.

BMI, body mass index; z-BMI, body mass index z score; WC, waist circumference; WHtR, weight to height ratio; TPI, Triponderal mass index; SBP, systolic blood pressure; DBP, diastolic blood pressure.

The bold values indicate statistically significant results with *p*<0.05.

When comparing the anthropometric measurements, the NAFPD group showed significantly higher values in weight, BMI, WC, WHtR, and TPI (*p* < 0.001). Similarly, subcutaneous and visceral fat measurements were significantly higher in the NAFPD group (*p* < 0.001). Regarding blood pressure measurements, SBP was significantly higher in the NAFPD group (*p* = 0.009). However, there was no significant difference in DBP between the two groups (*p* = 0.059). Additionally, liver steatosis was more prevalent in the NAFPD group (63.3 %) than in the control group (5.2 %) (*p* < 0.001).

### Logistic regression models

The study results, adjusting for age and sex, as well as age, sex, and z-BMI, are presented in Table 3.

**Table 3:** Logistic regression models.

	Adjusted by age and sex		Adjusted by age, sex and z - BMI	
	OR (95 % CI)	*p*-value	OR (95 % CI)	*p*-value
z- BMI	4.3 (2.71–6.82)	**<0.001**		
WC	1.13 (1.09–1.17)	**<0.001**	1.09 (1.01–1.17)	**0.022**
WHtR	1.23 (1.16–1.3)	**<0.001**	1.13 (1–1.27)	0.053
TPI	1.61 (1–4–1.86)	**<0.001**	1.28 (0.8–2.07)	0.306
SBP	1.06 (1.02–1.11)	**0.002**	1.03 (0.98–1.07)	0.212
DBP	1.07 (1.02–1.12)	**0.01**	1.05 (0.99–1.11)	0.083
Subcutaneous fat	1.09 (1.06–1.12)	**<0.001**	1.04 (1–1.08)	**0.029**
Visceral fat	1.07 (1.05–1.1)	**<0.001**	1.03 (1–1.06)	0.066
Liver steatosis	34.37 (14.81–79.77)	**<0.001**	13.02 (5.07–33.45)	**<0.001**

z-BMI, body mass index z score; WC, waist circumference; WHtR, weight to height ratio; TPI, Triponderal mass index; SBP, systolic blood pressure; DBP, diastolic blood pressure.

The bold values indicate statistically significant results with *p*<0.05.

*Adjusted for age and sex:* All anthropometric and adiposity markers exhibited strong associations with NAFPD: BMI had an OR of 4.3 (2.71–6.82, *p* < 0.001), WC an OR of 1.13 (1.09–1.17, *p* < 0.001), WHtR had an OR of 1.23 (1.16–1.3, *p* < 0.001), and the TPI an OR of 1.61 (1.4–1.86, *p* < 0.001). Additionally, SBP and DBP also showed significant associations, with ORs of 1.06 (1.02–1.11, *p* = 0.002) and 1.07 (1.02–1.12, *p* = 0.01), respectively. Subcutaneous fat and visceral fat demonstrated significant associations with NAFPD, with ORs of 1.09 (1.06–1.12, *p* < 0.001) and 1.07 (1.05–1.1, *p* < 0.001), respectively.

*Adjusted for age, sex, and z-BMI:* After additional adjustment for z-BMI, the association between WC and NAFPD remained significant, with an OR of 1.09 (*p* = 0.022). WHtR exhibited a borderline association, with an OR of 1.13 (*p* = 0.053), while the TPI showed no significant association. SBP, DBP, subcutaneous fat, and visceral fat did not maintain significant associations after adjusting for age, sex, and z-BMI.

Liver steatosis exhibited a remarkably strong association with NAFPD in both models, with an OR of 34.37 (*p* < 0.001) in the age and sex-adjusted model and an OR of 13.02 (*p* < 0.001) in the model further adjusted for z-BMI.

## Discussion

The present findings revealed a 4 % prevalence of NAFPD in adolescents of 15.43 years. Individuals with NAFPD in the cohort displayed distinctive anthropometric characteristics, elevated blood pressure, and increased subcutaneous and visceral fat compared to those without fatty pancreas. Furthermore, z-BMI, WC, and weight-to-height ratio remained strongly associated with NAFPD in adolescents even after adjusting for age and sex. Although other anthropometric measurements exhibited significant associations in models adjusted for sex and age, these associations did not maintain significance after BMI adjustment. Remarkably, this study revealed a robust association between liver steatosis and NAFPD in adolescents.

Transabdominal ultrasound is a rapid, cost-effective, and safe method, but it lacks sensitivity for detecting mild to moderate fatty infiltration of the pancreas and may not consistently visualize this organ, particularly in patients with obesity.^15^ This modality is operator-dependent, and the subjective comparison of pancreatic echogenicity to hepatic or nephrotic echogenicity introduces variability.^16^ Despite these limitations, the US remains widely used for FP detection, primarily due to its easy accessibility, cost-effectiveness, and the absence of complications associated with its implementation.

To our knowledge, this is the first study aiming to determine the prevalence of NAFPD in the general population of adolescents. The authors found a prevalence of NAFPD of 4 %, reaching almost 20 % in participants with obesity. It is important to highlight that there was a percentage of children in which the pancreas was not visualized (5.5 % of the total sample), but they did have US findings compatible with LS (44.2 % of those in whom the pancreas was not visualized). Therefore, and considering the strong association between LS and NAFPD, the authors believe that the prevalence of fatty pancreas may be underestimated. A systematic review published in 2023, showed a bidirectional relationship between fatty pancreas and LS, with LS associated with a 6.18-fold increased risk of fatty pancreas and fatty pancreas linked to a 9.56-fold increased risk of LS. Additionally, a transabdominal ultrasound revealed a higher likelihood of severe LS in patients with a fatty pancreas, and the coexistence of a fatty pancreas was linked to an increased risk of Non-Alcoholic SteatoHepatitis (NASH) and advanced fibrosis in LS patients.^17^ The authors should also mention that it is possible that the observed prevalence of NAFPD in this study may not accurately represent the true prevalence due to the limited sensitivity of the ultrasound method.

Notably, the prevalence of NAFPD in pediatrics remains ambiguous. In 2016, Pham et al. conducted a study to assess the prevalence of NAFPD in 232 patients 2 to 18 years old, which was found to be 10 %. However, this result may not be representative of the general pediatric population since the study was performed in hospitalized patients.^18^ In Asian adult populations, prevalence data has been reported to range from 16 % to 35 % in various studies.^19^^,^^20^

Obesity is considered the most significant risk factor for developing NAFPD. This association was initially proposed by Ogilvie and has been consistently validated in subsequent studies.^12^^,^^21^^,^^22^ The present study supports this association, revealing that adolescents with NAFPD exhibited higher z-BMI compared to controls. This finding concurs with prior human studies utilizing autopsy assessments or various imaging modalities like US, computed tomography, or MRI.^23^^,^^24^ It is important to highlight that the majority of previous studies have been conducted in the adult population, and gaining insight into the prevalence of NAFPD at earlier developmental stages could potentially enable interventions aimed at improving the prognosis of this condition.

Numerous surrogate indicators of visceral adiposity, such as WC, WHtR, and TPI, have been explored extensively. Various studies have demonstrated their correlation with body fat mass and visceral adiposity, employing diverse methodologies in both children with obesity and adults.^25^^,^^26^ In the comparative analysis of anthropometric measurements, the NAFPD group demonstrated significantly heightened values in WC, WHtR, and TPI. Subcutaneous and visceral fat measurements were also notably elevated in the NAFPD group. These results suggest that adolescents with fatty pancreas exhibit increased central adiposity and elevated levels of subcutaneous and visceral fat, suggesting a potential link between pancreatic fat accumulation and overall body fat distribution. Moreover, these findings, adjusting for age and sex, revealed a significant association between WC and NAFPD. Importantly, this association remained significant even after adjusting for z-BMI, indicating that WC might independently contribute to NAFPD development beyond its correlation with BMI.

Metabolic syndrome (MetS), characterized by abdominal obesity, insulin resistance, hypertension, and hyperlipidemia, poses an increased risk of cardiovascular diseases.^27^ Evidence increasingly links NAFPD with all MetS components in adolescents and adults.^12^^,^^28^^,^^29^ Chiyanika et al.^30^ published in 2019 a report that describes the relationship between NAFPD, body fat, and the risk of metabolic syndrome in 52 Chinese adolescents (14–18 years) with both obesity and LS. They found that 50 % had NAFPD, 38 % had metabolic syndrome, and 81 % exhibited insulin resistance. NAFPD in obesity was associated with metabolic syndrome (OR = 1.70). Although the sample lacked all the elements for diagnosing metabolic syndrome, the authors had to include two components: waist circumference and blood pressure. Notably, a robust association with NAFPD, independent of BMI, was observed for waist circumference. Additionally, blood pressure was elevated in the NAFPD group compared to controls, though not reaching hypertensive levels. These elevated readings may suggest a potential predisposition to hypertension in subsequent stages.

As mentioned above, recent studies have demonstrated a significant correlation between NAFPD and LS. In a prospective study involving 293 patients, it was found that 68 % of individuals with NAFPD also had LS. Furthermore, nearly all subjects (97 %) with LS were found to have NAFPD as well.^12^ These findings strongly indicate a potential physiopathological link between the two conditions.^21^ Della Corte et al.^4^ evaluated 121 pediatric patients with echogenic-demonstrated LS, identifying 58 patients with NAFPD. The NAFPD group exhibited notably higher z-BMI, fasting insulin levels, and HOMA-IR. Moreover, they displayed a more advanced liver disease phenotype, characterized by elevated values of fibrosis, ballooning, and NAFLD Activity Score, compared to the group without NAFPD. These results suggest a close relationship between NAFPD and the severity of liver disease in pediatric patients with LS.

The strengths of the present study include the representativeness of the adolescent population, the high number of participants, and that it is one of the few studies that provide data on the fatty pancreas in the general population of adolescents.

This study has some limitations. Firstly, the diagnosis of NAFPD was based on US rather than MRI, which is currently acknowledged as the most accurate imaging modality for measuring pancreatic fat content. The use of the US was driven by challenges in accessing MRI, primarily due to its high cost. While the US is the most commonly used non-invasive tool for abdominal imaging, its limitations include difficulties in achieving clear visualization of the pancreas, especially in individuals with obesity. The operator-dependent and subjective nature of the US further complicates its effectiveness. However, several authors have advocated the abdominal US as a reliable screening tool for diagnosing pancreatic conditions, given its significant accuracy, cost-effectiveness, and nonside effects. Additionally, alternative diagnostic tools such as computed tomography (CT) and MRI offer higher accuracy in quantifying pancreatic fat and could be considered in future research to address these limitations. The study lacks measurements of inter- and intra-observer variability, which could have provided insights into the reliability and consistency of the results. Another limitation is the imbalance between the number of NAFPD cases and the control group, which may affect the robustness of some of these analyses. Future studies with a larger and more balanced sample will be necessary to further validate the present results. The absence of biochemical data, such as glycemia, insulin and lipid profiles, represents a limitation, preventing a more comprehensive description of metabolic alterations in adolescents with NAFPD.

## Conclusions

In the Chilean adolescent population, the prevalence of NAFPD is 4 %. Adolescents with obesity exhibit a higher accumulation of pancreatic fat compared to non-obese adolescents. Individuals with NAFPD display distinct anthropometric characteristics, higher blood pressure, and increased subcutaneous and visceral fat in comparison to those without fatty pancreas. NAFPD is strongly associated with WC and LS.

## Authors’ contributions

All the included authors have made substantial contributions to this work. Specifically, GA drafted the manuscript with the assistance of JCG. JCG obtained funding for the abdominal US and the anthropometric data collection of the GOCS cohort. FDB and CG performed the abdominal US. LV, GA and JCG conducted the interpretation of the data. GA, JCG, AP, LV and TC contributed to the conceptualization of the study. All authors critically reviewed the manuscript and approved the final version.

## Source of funding

This study was supported by ‘‘Fondo Nacional de Desarrollo Científico y Tecnológico (FONDECYT)’’ 11190856 and 1200839.

## Conflicts of interest

All authors declare that there is no conflict of interest that could be perceived as prejudicing the impartiality of the research reported.
